# Supplementary material for: What does it take to design digitally enabled performance management and incentive interventions for community health programs: Lessons from Ethiopia
Source: PLOS Digit Health. 2025 Jul 3;4(7):e0000914. doi: 10.1371/journal.pdig.0000914 (PMC12225878; doi:10.1371/journal.pdig.0000914)
Supplement: S1 Table — (DOCX) [file pdig.0000914.s001.docx]

S1_Table: Key Performance Indicators (KPIs) for measuring the performance of HEWs, HPs and supervisors

| Health post KPI | |
| --- | --- |
| *1* | Contraceptive acceptance rate |
| *2* | Proportion of antenatal care (ANC) 4^th^ visit |
| *3* | Immunization dropout rate from Pentavalent 1 - Pentavalent 3 |
| *4* | Proportion of under two years children participated in growth monitoring and promotion |
| *5* | Proportion of pregnant and lactating women nutrition screening for acute malnutrition |
| *6* | Proportion of model Households |
| health extension workers (HEW) KPI | |
| 1 | # of clients who received modern family planning service by health extension workers (HEW) |
| 2 | # of pregnant women referred by HEWs for ANC 4^th^ visit |
| 3 | # of  pregnant women referred by HEWs for ANC 1^st^ visit |
| 4 | # of children immunized for Pentavalent 1 |
| *5* | # of children immunized for Pentavalent 3 |
| 6 | # of children < 1yrs of age fully immunized |
| 7 | # of pregnant and lactating women screened for malnutrition. |
| 8 | # of children under 2 years weighted (screened) during growth monitoring and promotion session |
| 9 | # of household member registered using eCHIS |
| 10 | # of model households |
| Supervisor KPI | |
| 1 | # of essential drug available in the health posts |
| 2 | # of essential medical equipment available in the health posts |
| 3 | # of supportive supervision provided using standard checklist per month |
| 4 | # of referrals (disaggregated by status: Complete, Referral + Feedback, Referral) |
| 5 | # of review meeting conducted at the health center per month |
| 5 | Health posts average KPIs score |
