# Supplementary material for: What does it take to design digitally enabled performance management and incentive interventions for community health programs: Lessons from Ethiopia
Source: PLOS Digit Health. 2025 Jul 3;4(7):e0000914. doi: 10.1371/journal.pdig.0000914 (PMC12225878; doi:10.1371/journal.pdig.0000914)
Supplement: Attachment — Submitted filename: Point by point response to Review Comments.docx [file pdig.0000914.s003.docx]

**Point by point response to Review Comments**

Reviewer 1:

1. Introduction: The problem statement is strong, but could include more references to previous digital health PM/PBI interventions in similar settings.

*Response: Thank you for your suggestions. We have added more references in the introduction/problem statement section. Please see on page 5 from line number 75 to 93, and on page 7, 121-127*

2. Methodology Section: Needs more clarity and rigor. The study lacks explicit methodological details about study participants and data collection methods (on how data was collected, analyzed, and validated).

*Response: Thank you for your comments. We have reviewed the methods section and indicated the study participants and data collection methods for the Landscape review and HCD. Please see page 10 from line number 185-200. This manuscript is intended to highlight the approaches and processes we followed in designing the digitally enabled PM/PBI interventions. The follow-on pilot testing of the designed PM/PBI interventions could have explicit methodology in selecting participants, data collection, and analysis procedures.*

3. Results & Discussion: Reduce redundancy—many points in the discussion are already covered in the results.
   Add a deeper analysis of challenges and feasibility. Discuss how the Ethiopian context may influence the scalability of this model. Add a comparative discussion with similar digital health interventions from other low-resource settings.

*Response: Thank you for your invaluable comments and suggestions. We have reduced redundancies by removing redundant sections from the discussion. The whole discussion section has been reviewed in light of the given comments and suggestions. Please see the revisions in the discussion section, from page 20 to 27, line numbers 404 to 563.*

*We have made a deeper analysis of the challenges, feasibility, salability, and sustainability, making a comparative discussion with similar digital health interventions from other low-resource settings. Please see pages 24 to 27; line numbers 490 to 563*

4. Conduct a thorough grammatical review and restructuring to remove redundancy, grammatical errors and improve clarity.

*Response: Thank you for your comment. We have done a thorough grammatical review of the whole manuscript and removed redundancies, grammatical errors, and improved clarity. Please see the full manuscript.*
